# Ginsenoside Rg1 Reduced Microglial Activation and Mitochondrial Dysfunction to Alleviate Depression-Like Behaviour Via the GAS5/EZH2/SOCS3/NRF2 Axis

**DOI:** 10.1007/s12035-022-02740-7

**Published:** 2022-03-01

**Authors:** Junnan Li, Wei Gao, Zhonghui Zhao, Yannan Li, Lixuan Yang, Wei Wei, Feifei Ren, Yang Li, Yao Yu, Wenzhe Duan, Jingchun Li, Baoan Dai, Rongjuan Guo

**Affiliations:** 1 Second Clinical Medical College, Beijing University of Chinese Medicine, Beijing, 100029 People’s Republic of China; 2 Tsinghua University, Yuquan Hospital, Beijing, 100040 People’s Republic of China; 3 Beijing Hospital of Traditional Chinese Medicine, Capital Medical University, Beijing, 100010 People’s Republic of China; 4 Beijing Changping Hospital of Integrated Chinese and Western Medicine, Beijing, 102208 People’s Republic of China; 5 Department of Neurology, Dongfang Hospital Beijing University of Chinese Medicine, No. 6 Fangxingyuan 1st Block, Fengtai District, Beijing, 100078 People’s Republic of China

**Keywords:** Ginsenoside Rg1, GAS5, EZH2, Depression, SOCS3, NRF2

## Abstract

Ginsenoside Rg1 is the principal active ingredient in ginseng. The antidepressant effects of Rg1 have been validated; however, the specific underlying mechanism of this effect needs further research. Rats were subjected to the chronic restraint stress (CRS) depression model. Rg1, or a positive control drug, was administered to the rats. Depression-like behaviours were evaluated through behavioural experiments. Cytokine, mRNA, protein, ATP, and mitochondria DNA levels were detected using the indicated methods. Lentivirus-packaged plasmids were injected into the rat brain for GAS5 overexpression or knockdown. In vitro mitochondrial dysfunction was evaluated by detecting mitochondrial reactive oxygen species and mitochondrial membrane potential. Direct interaction between GAS5 and EZH2 was validated by RNA immunoprecipitation and RNA pull-down assay. The enrichment of EZH2 and H3K27me3 was evaluated through chromatin immunoprecipitation quantitative real-time PCR. Rg1 treatment alleviated depression-like behaviours, microglial activation, and mitochondrial dysfunction in CRS rats. Similarly, GAS5 knockdown revealed a similar protective effect of Rg1 treatment. GAS5 overexpression in the rat brain compromised the protective effect of Rg1 treatment. Moreover, Rg1 treatment or GAS5 knockdown attenuated microglial activation and mitochondrial dysfunction in vitro. Mechanically, GAS5 was suppressed SOCS3 and NRF2 expression by facilitating EZH2-mediated transcriptional repression. Rg1 attenuated microglial activation and improved mitochondrial dysfunction in depression by downregulating GAS5 expression. Mechanically, GAS5 might regulate microglial activation and mitochondrial dysfunction via the epigenetic suppression of NRF2 and SOCS3.

## Introduction

Depression is a type of emotional disorder that brings a tremendous burden to society and individuals. Depression is characterized by high morbidity, mortality, and disability rates [1]. Typical symptoms of depression include depressed mood, retardation of thought, loss of volitional activity, and cognitive impairment [2]. So far, the cause of depression remains elusive. Several biological, psychological, and social environmental factors are associated with the pathogenesis of depression. Biological factors mainly involve heredity, neurobiochemical, neuroendocrine, and nerve regeneration [3]. Currently, most antidepressants in clinical use are synthetic compounds, including selective 5-HT reuptake inhibitors (SSRIs) [4]. Although the current treatments improve depression, they fail to resolve the symptoms entirely in more than half of the cases. Thus, it is urgent to identify novel potential candidates for the treatment of depression.

Microglia, a type of glial cell, are macrophages in the brain and spinal cord. As the primary immune line of defense in the central nervous system (CNS), microglia clears the damaged nerves and infectious substances in the CNS [5]. However, overactivated microglia can cause neurotoxicity by releasing pro-inflammatory cytokines [6]. Several studies have reported that the overactivation of microglia plays a crucial role in the pathogenesis of neurodegenerative diseases, including Parkinson’s and Alzheimer’s diseases [7, 8]. Notably, microglia are involved in the pathogenesis of depression [9]. Overactivation of microglia and upregulation of inflammatory cytokines have been observed in depression models [10]. Moreover, growing evidence has indicated that inhibiting the overactivation of microglia can ameliorate depression-like behaviour. Bassett et al. reported that minocycline attenuated depression-like behaviour in chronic mild stress (CMS)-induced mice via suppressing the overactivation of microglia [11]. Melatonin can also attenuate depression-like behaviours by inhibiting microglial activation [12]. Hence, targeting microglial activation may be an effective manner to treat depression.

Mitochondria are the principal organelles of cellular energy metabolism and reactive oxygen species (ROS) generation [13]. Recently, an increasing number of studies have reported that mitochondrial function and structure are abnormal in patients with depression, and changes in the mitochondrial ultrastructure can lead to energy metabolism disorders. Therefore, mitochondrial dysfunction is proposed to be involved in the pathogenesis of depression [14]. Depression is usually associated with an increase in intracellular ROS levels and a decrease in antioxidant capacity. As the primary production site of cellular ROS, mitochondria are sensitive to oxidative stress injury. Mitochondrial dysfunction can further aggravate oxidative damage [15]. A study by Wang et al. suggested that injecting isolated mitochondria abrogated microglial activation, suppressed ROS generation, and ameliorated depression-like behaviours [16]. Hence, discovering the significance of mitochondrial dysfunction in the pathogenesis of depression might help generate novel therapeutic targets for depression treatment.

Ginsenoside Rg1 is a steroid compound, also known as triterpenoid saponin, primarily found in *Panax ginseng* and is regarded as the active ingredient in ginseng. Numerous studies have demonstrated the protective role of Rg1 in neurological diseases, including cerebral ischemia, Alzheimer’s disease, and Parkinson’s disease [17–19]. Additionally, growing evidence has revealed the protective role of Rg1 in depression. Rg1 reportedly abrogates oxidative stress and inflammation in the neurons to alleviate stress-induced depression-like behaviours [20]. Another study reported that Rg1 activates the BDNF signaling pathway and promotes neurogenesis [21].

Moreover, a study by Yu et al. revealed that Rg1 might exert its protective role in depression via the regulation of neuronal structural plasticity [22]. Although the functional role of Rg1 has been demonstrated in depression, it remains unclear whether Rg1 could exhibit its protective effect by improving mitochondrial function. In addition, the underlying molecular mechanism of Rg1 in depression also remains largely unknown.

Long non-coding RNAs (lncRNAs) are RNAs with more than 200 bases in length that cannot encode proteins [23]. Recently, lncRNAs have been demonstrated to play a crucial role in the pathogenesis of depression. A study on the analysis of lncRNA expression in peripheral blood leukocytes of patients with depression revealed that lncRNA RMRP in the patients was significantly reduced, which might be used as a depression marker [24]. A functional study revealed that lncRNA uc.80 switches microglia polarization to M2 subtype and ameliorate depression-like behaviours in rats [25]. Additionally, lncRNA MIR155HG inhibits depression-like behaviours by upregulating BDNF expression [26]. lncRNA growth arresting-specific 5 (GAS5) has been proven to be involved in various physiological and pathological processes. Specifically, inhibiting lncRNA GAS5 can improve depression-like behaviours by reducing neuronal damage in the hippocampus [27]. However, the underlying mechanism must be further investigated.

In the present study, we aimed to verify the protective role of Rg1 in depression. Rg1 attenuated microglial activation and improved mitochondrial dysfunction by downregulating the expression of GAS5. The protective role of GAS5 inhibition was also proven in the depression model. Mechanically, GAS5 regulated microglial activation and mitochondrial dysfunction via the epigenetic suppression of NRF2 and SOCS3. Our result might provide novel insight for understanding the role of Rg1 in the treatment of depression.

## Materials and Methods

### Animals

Male SD rats (weight: 200–250 g) were purchased from Charles River Laboratories (Beijing, China) and maintained at 22℃ ± 1℃ at a diurnal cycle of 12-h and were provided with free access to water and food. All animal experiments were conducted in strict adherence to the China-Japan Friendship Hospital guidelines on the care and use of laboratory animals. The study was approved by the Ethics Committee of China-Japan Friendship Hospital (Approval number: zryhyy21-20-09-9).

### Plasmid Construction and Transfection

Sh-GAS5 and the negative-control shRNA (sh-NC) were synthesized by GenePharma (Shanghai, China), and cloned into a pLKO.1 vector. The pLKO.1-shGAS5 plasmids were then transfected into HEK293T cells with a psPAX2 packaging plasmid using the Lipofectamine 3000 to produce shRNA-containing lentivirus (lenti-shRNA). The lenti-shRNA was then used to infect cells or microinjected into an animal ventricle to construct GAS5 silent rats.

A GAS5-overexpressing plasmid was generated using a full-length GAS5 that was cloned and inserted into multiple cloning sites of the pLenti6.3/V5-DEST expression plasmid and then transfected into 293 T cells using the Lipofectamine 3000, together with pLP1 and pLP2 packaging plasmids and pLP/VSVG envelope plasmid, to produce GAS5-expressing lentivirus. The resultant lentivirus was used to generate GAS5-overexpressing (lenti-GAS5) rats or cells.

### Chronic Restraint Stress Model

Before chronic restraint stress (CRS) modeling, 1 week (days 1–3: adaptation to the environment; days 4–5: 2 bottles of pure water; days 6–7: a bottle of 1% sucrose water and a bottle of fresh water) was allowed for adaptation purpose. The rats in CRS group were then restricted in an adjustable cylindrical plastic bound pipe (made by 50 ml centrifuge tubes with pre-blistered air holes dispersed throughout the tube and a small hole in the center of the cap through which the mouse’s tail could be exposed to the air) fitted to allow them to breathe for 4 h every day (from 09:00 to 13:00) with no food and water for 28 consecutive days. A group of rats (control) was not subjected to any treatment other than restraining food and water supply during 09:00–13:00 to match the test rats.

**Fig. 1:**
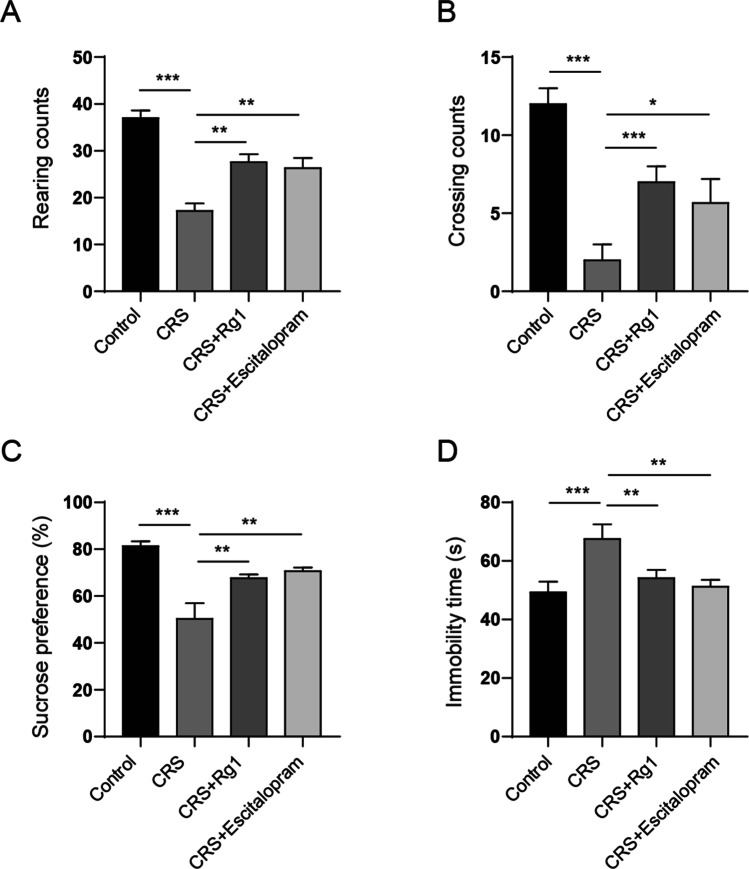
Rg1 ameliorated depression-like behaviours in CRS-induced rats. CRS rats were restricted in an adjustable cylindrical plastic bound pipe fitted to allow them to breathe for 4 h every day (from 09:00 to 13:00) with no food and water for 28 consecutive days. A group of rats (control) was not subjected to any treatment other than restraining food and water supply during 09:00–13:00. Rg1 (20 mg/kg/day) or escitalopram (1 mg/kg/day) was administered to the rats via the intragastric route 1 h after CRS every day. After 24 h accommodation, behaviours tests were performed. **A** and **B** Rearing and crossing counts in open field test. **C** Sucrose preference test. **D** Immobility time in forced swim test. *P < 0.05; **P < 0.01; ***P < 0.001

**Fig. 2:**
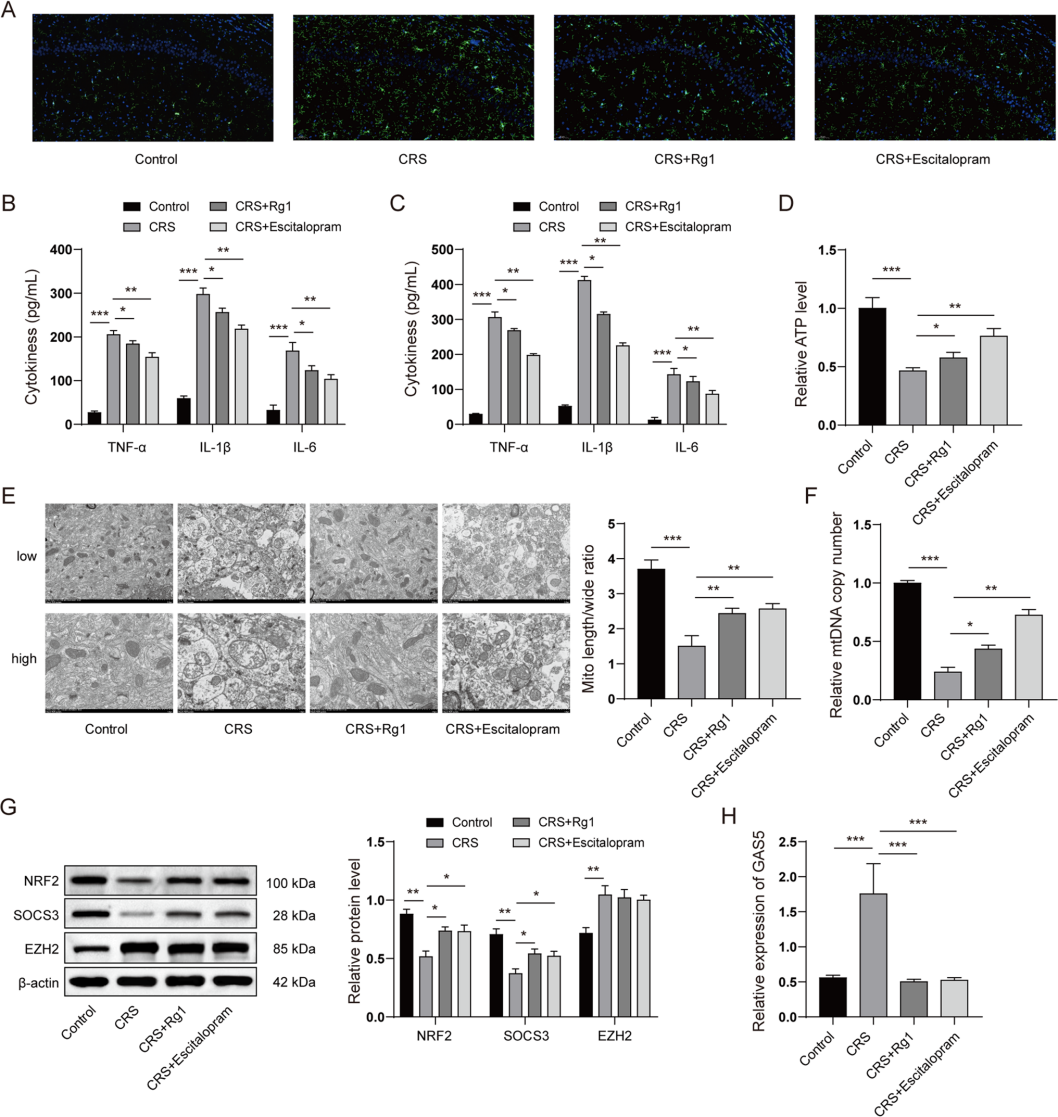
Rg1 abrogated microglial activation and attenuated mitochondrial dysfunction in CRS-induced rats. CRS rats were restricted in an adjustable cylindrical plastic bound pipe fitted to allow them to breathe for 4 h every day (from 09:00 to 13:00) with no food and water for 28 consecutive days. A group of rats (control) was not subjected to any treatment other than restraining food and water supply during 09:00–13:00. Rg1 (20 mg/kg/day) or escitalopram (1 mg/kg/day) was administered to the rats via the intragastric route 1 h after CRS every day. Hippocampus and serum were collected. **A** IBA-1 positive cells in the hippocampus were detected by immunofluorescence. **B** and **C** Pro-inflammatory cytokines (TNF-α, IL-1β, and IL-6) in the serum (**B**) and hippocampus (**C**) were detected by ELISA. **D** ATP level in hippocampus was detected. **E** The morphology of mitochondria in hippocampus was observed by TEM. **F** The copy number of mitochondrial DNA (mtDNA) in hippocampus. **G** The protein levels of SOCS3, NRF2, and EZH2 in hippocampus were detected by western blots. **H** The level of GAS5 was detected by qPCR. *P < 0.05; **P < 0.01; ***P < 0.001

**Fig. 3:**
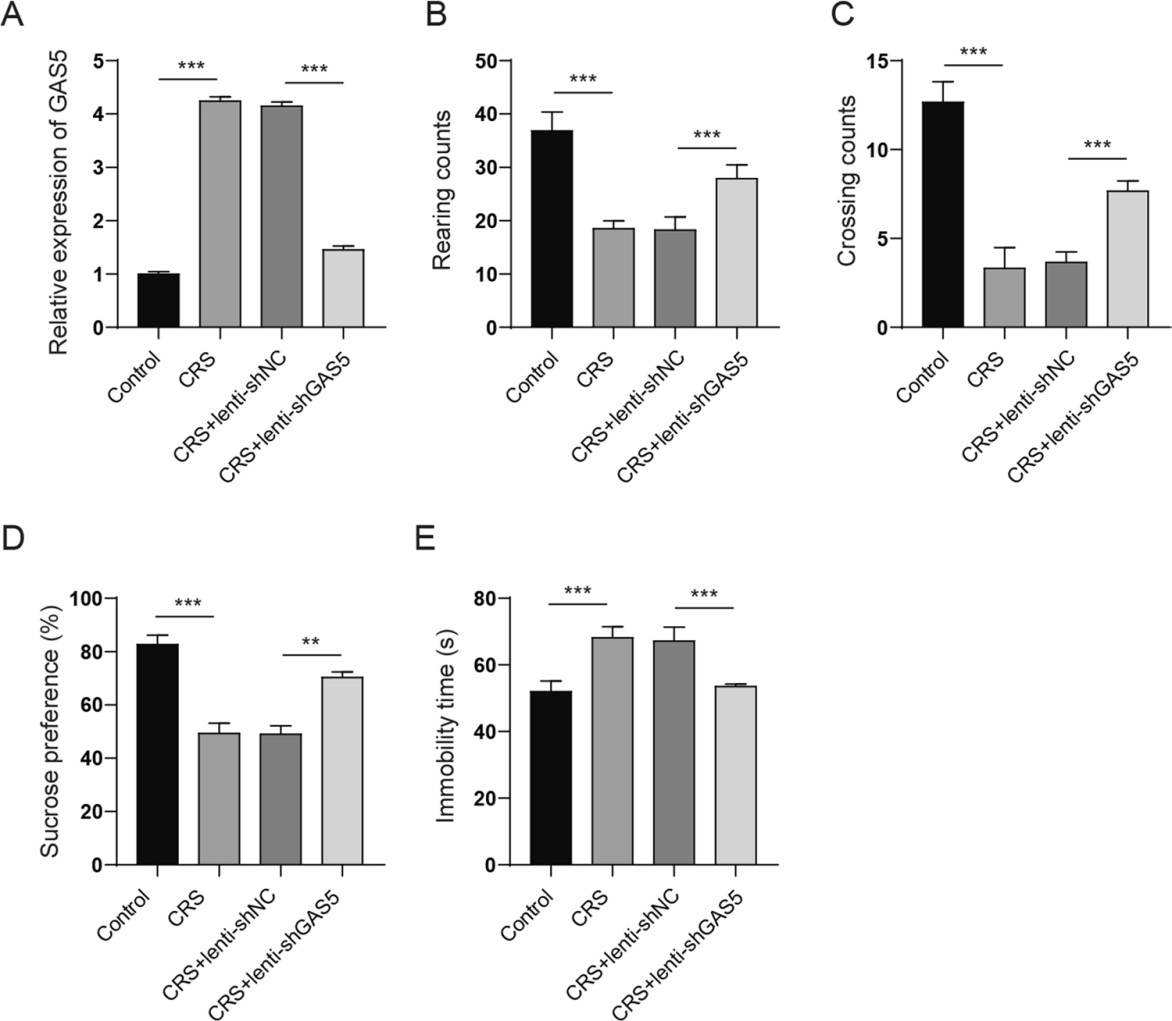
GAS knockdown ameliorated depression-like behaviours in CRS-induced rats. In vivo GAS5 knockdown was achieved by injecting lentivirus-packaged GAS5 shRNA (Lenti-shGAS5) into the lateral ventricle at the following coordinates: 0.3 mm behind the bregma and 1.0 mm lateral from the sagittal midline, at a depth of 2.2 mm from the skull surface on the last day of adaptation by using a Nanoject II (Drummond) system at a rate of 0.1 μl per min.. Forty-eight hours after in vivo injection, the rats were subjected to CRS stimulation for 28 days. After 24 h accommodation, behaviours tests were performed. **A** The GAS5 level in hippocampus was detected by qPCR. **B** and **C** Rearing and crossing counts in the open field test. **D** Sucrose preference test. **E** Immobility time in the forced swim test. *P < 0.05; **P < 0.01; ***P < 0.001

**Fig. 4:**
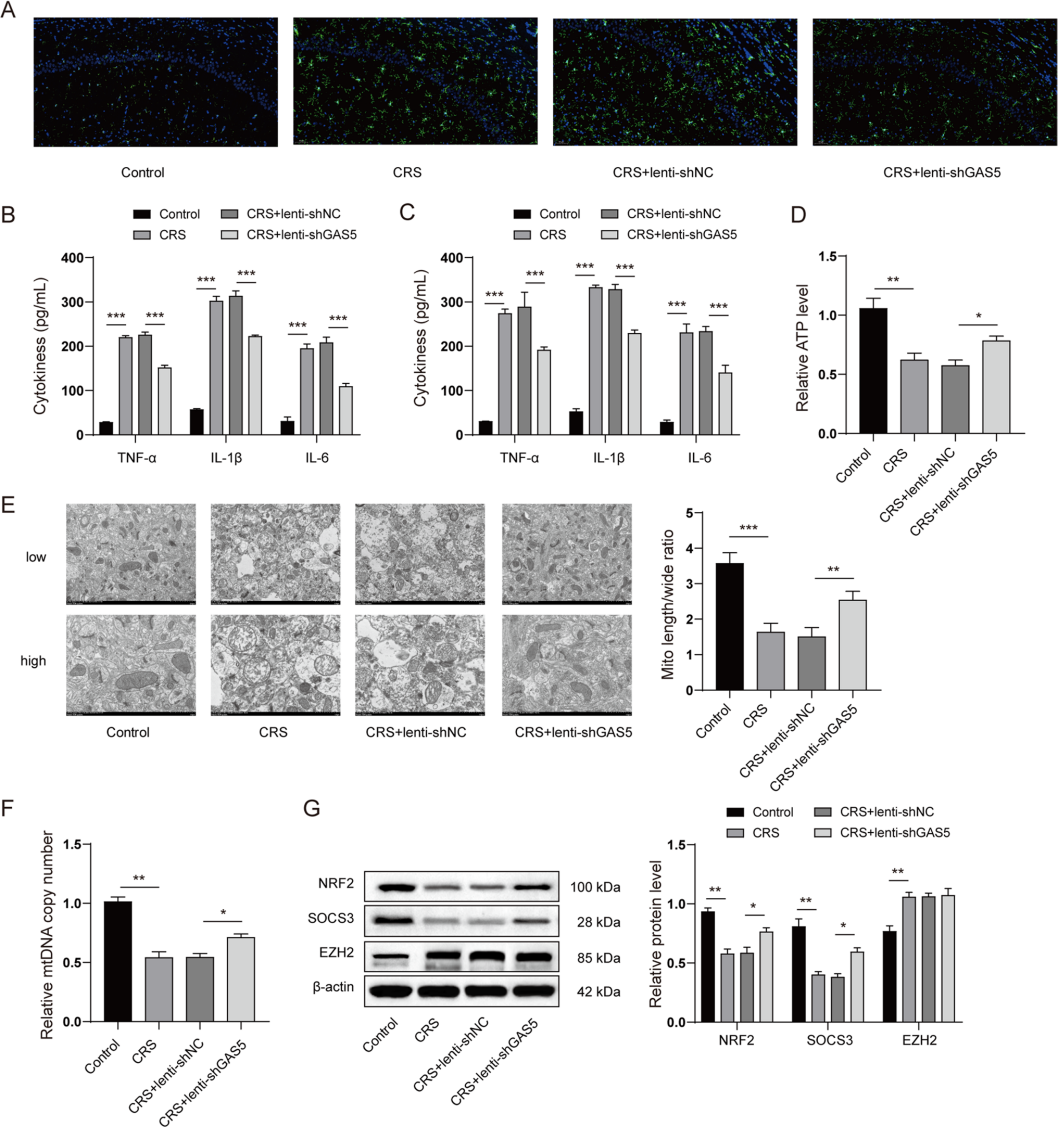
GAS5 knockdown suppressed microglial activation and attenuated mitochondrial dysfunction in CRS-induced rats. In vivo GAS5 knockdown was achieved by injecting lentivirus-packaged GAS5 shRNA (Lenti-shGAS5) into the lateral ventricle at the following coordinates: 0.3 mm behind the bregma and 1.0 mm lateral from the sagittal midline, at a depth of 2.2 mm from the skull surface on the last day of adaptation by using a Nanoject II (Drummond) system at a rate of 0.1 μl per min. Forty-eight hours after in vivo injection, the rats were subjected to CRS stimulation for 28 days. Hippocampus and serum were collected. **A** IBA-1 positive cells in the hippocampus were detected by immunofluorescence. **B** and **C** The levels of pro-inflammatory cytokines (TNF-α, IL-1β, and IL-6) in the serum (**B**) and hippocampus (**C**) were detected by ELISA. **D** ATP level in hippocampus was detected. **E** The morphology of mitochondria in hippocampus was observed by TEM. **F** The copy number of mitochondrial DNA (mtDNA) in hippocampus. **G** The protein levels of SOCS3, NRF2, and EZH2 in hippocampus were detected by western blots. *P < 0.05; **P < 0.01; ***P < 0.001

**Fig. 5:**
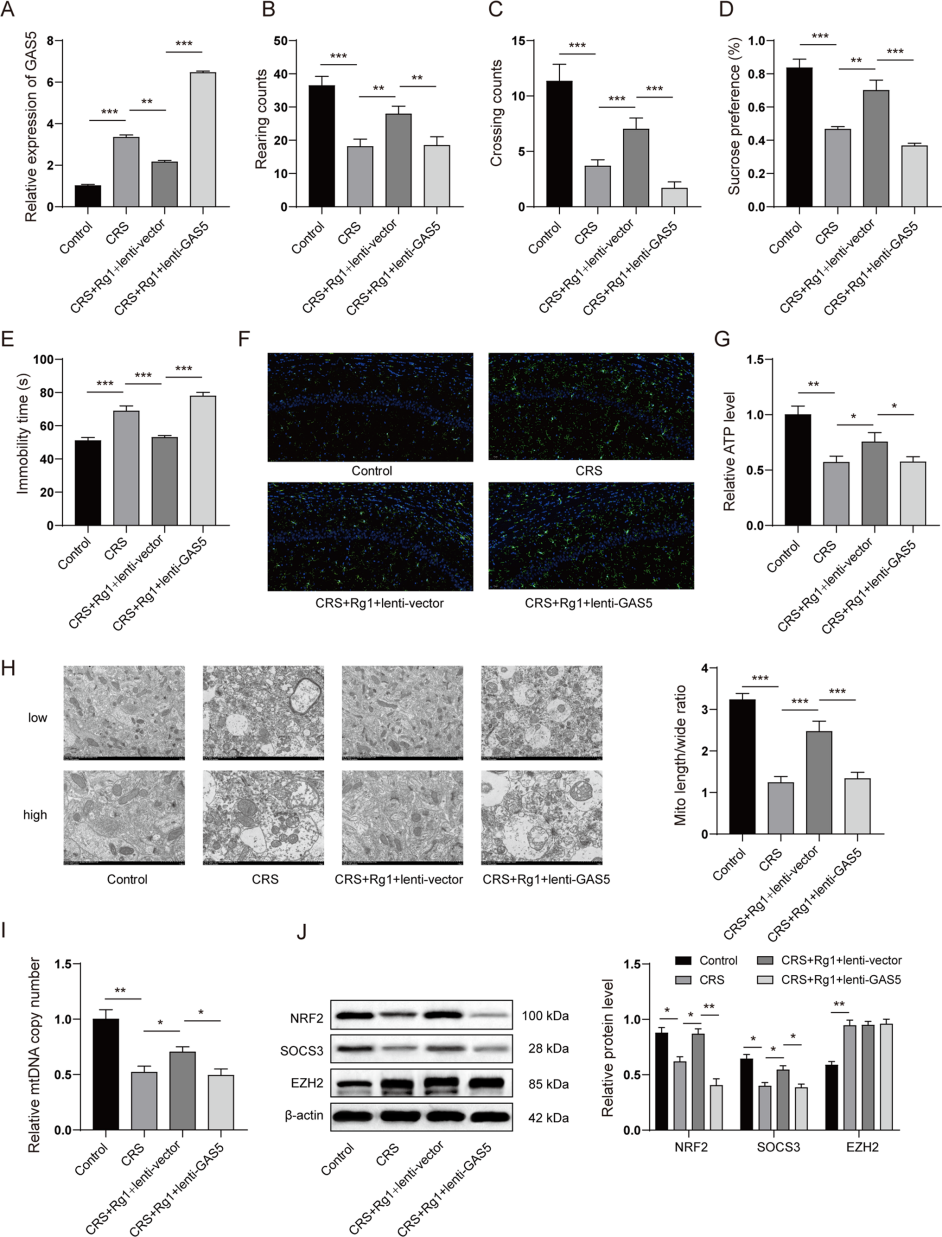
Rg1 exerted a protective effect on the rat CRS-induced depression model by downregulating GAS5. In vivo GAS5 knockdown was achieved by injecting lentivirus-packaged GAS5 shRNA (Lenti-shGAS5) into the lateral ventricle at the following coordinates: 0.3 mm behind the bregma and 1.0 mm lateral from the sagittal midline, at a depth of 2.2 mm from the skull surface on the last day of adaptation by using a Nanoject II (Drummond) system at a rate of 0.1 μl per min. Forty-eight hours after in vivo injection, rats were subjected to CRS stimulation for 28 days and Rg1 (20 mg/kg) treatment every day 1 h after CRS stimulation. 24 h after modeling, behaviour tests were performed. **A** The level of GAS5 in hippocampus was detected by qPCR. **B** and **C** Rearing and crossing counts in the open field test. **D** Sucrose preference test. **E** Immobility time in the forced swim test. **F** IBA-1 positive cells in the hippocampus were detected by immunofluorescence. **G** ATP level in hippocampus was detected. **H** The morphology of mitochondria in hippocampus was observed by TEM. **I** The copy number of mitochondrial DNA (mtDNA) in hippocampus. **J** The protein levels of SOCS3, NRF2, and EZH2 in hippocampus were detected by western blots. *P < 0.05; **P < 0.01; ***P < 0.001

The animal treatment (*n* ≥ 5) of the experiments is depicted in Figs. 1 and 2: for CRS + Rg1/escitalopram group, Rg1 (20 mg/kg/day) or escitalopram (1 mg/kg/day) was administered to the rats via the intragastric route 1 h after CRS. Meanwhile, an equal amount of fresh water was provided to the control and CRS group of rats. As depicted in Figs. 3 and 4, for lenti-shRNA injected group, rats were anesthetized with 5% chloral hydrate by intraperitoneal (i.p.) injection and placed on a stereotaxic apparatus. Small holes were drilled into the skull, and lenti-shRNA (5 μL of 10^8^ viral genome/μL; HANBIO, Shanghai, China) was microinjected into the lateral ventricle at the following coordinates: 0.3 mm behind the bregma and 1.0 mm lateral from the sagittal midline, at a depth of 2.2 mm from the skull surface on the last day of adaptation by using a Nanoject II (Drummond) system at a rate of 0.1 μl per min. The injection cannula was slowly withdrawn 5 min after the virus infusion. The scalp was then sealed, and injected mice were monitored as they recovered from anesthesia. Five microliter of normal saline was microinjected into the rats in the control and CRS groups, as mentioned above. As shown in Fig. 5, for the CRS + Rg1 + lenti-RNA group, the microinjection and Rg1 treatment methods were kept the same as mentioned above. Rats were recovered for 48 h before CRS modeling. Rg1 (HY-N0045) and escitalopram (HY-14258) were purchased from MedChemExpress.

### Cell Culture

The PC-12 cell line was purchased from the National Collection of Authenticated Cell Cultures (China, Shanghai) and the HAPI cell line from BeNa Culture Collection (Beijing, China). PC-12 cells were cultured in RPMI 1640 medium (Gibco, USA) and the HAPI cells in the DMEM/F-12 medium (Gibco). All culture systems were supplemented with 10% fetal bovine serum (FBS) and 1% penicillin–streptomycin (Gibco) under a humidified atmosphere of 5% CO_2_ at 37 °C.

**Fig. 6:**
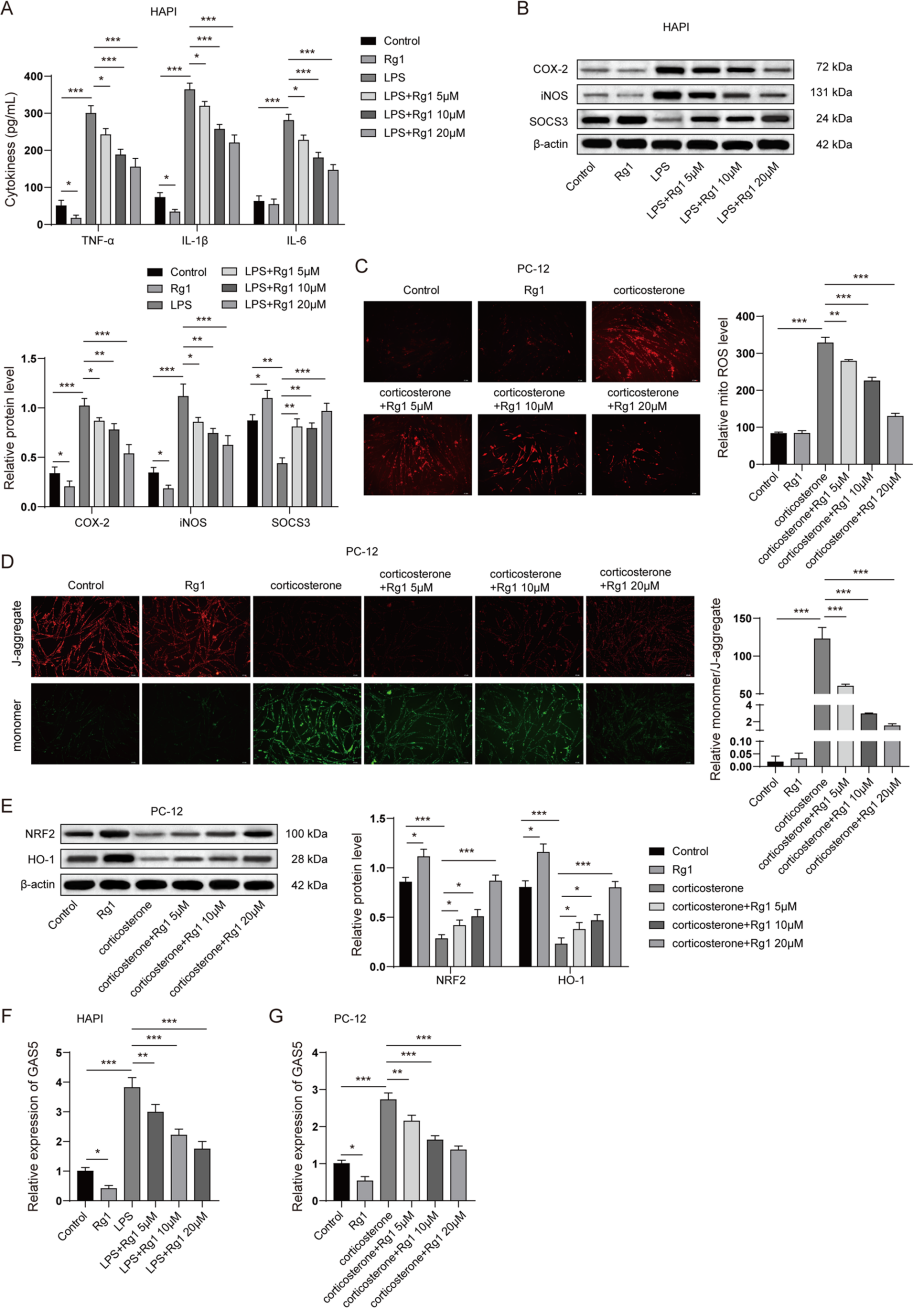
**Rg1 alleviated microglial activation and neuronal mitochondria dysfunction in vitro.** Rg1 alleviated microglial activation and neuronal mitochondria dysfunction in vitro. **A** and **B** Rat microglia cell line HAPI was stimulated with LPS (1 μM) or solution control for 2 h, followed by co-incubation with Rg1 (5 μM, 10 μM, 20 μM) for a total 24 h. **A** The release of pro-inflammatory cytokines in supernatant of HAPI cells was detected by ELISA. **B** The protein level of COX-2, iNOS, and SOCS3 in HAPI cells was detected by western blots. **C** to **E** PC-12 cells were stimulated with corticosterone (400 μM) or solution control for 2 h, followed by co-incubation with Rg1 (5 μM, 10 μM, 20 μM) for a total 24 h. **C** Mitochondrial ROS in PC-12 cells was detected by the mitoSOX kit. **D** Mitochondrial membrane potential in PC-12 cells was detected by the JC-1 assay. **E** The expression of NRF2 and HO-1 in PC-12 cells was detected by western blots. **F** Rat microglia cell line HAPI was stimulated with LPS (1 μM) or solution control for 2 h, followed by co-incubation with Rg1 (5 μM, 10 μM, 20 μM) for a total 24 h. The expression of GAS5 was detected by qRT-PCR. **G** PC-12 cells were stimulated with corticosterone (400 μM) or solution control for 2 h, followed by co-incubation with Rg1 (5 μM, 10 μM, 20 μM) for a total 24 h. The expression of GAS5 was detected by qRT-PCR. *P < 0.05; **P < 0.01; ***P < 0.001

**Fig. 7:**
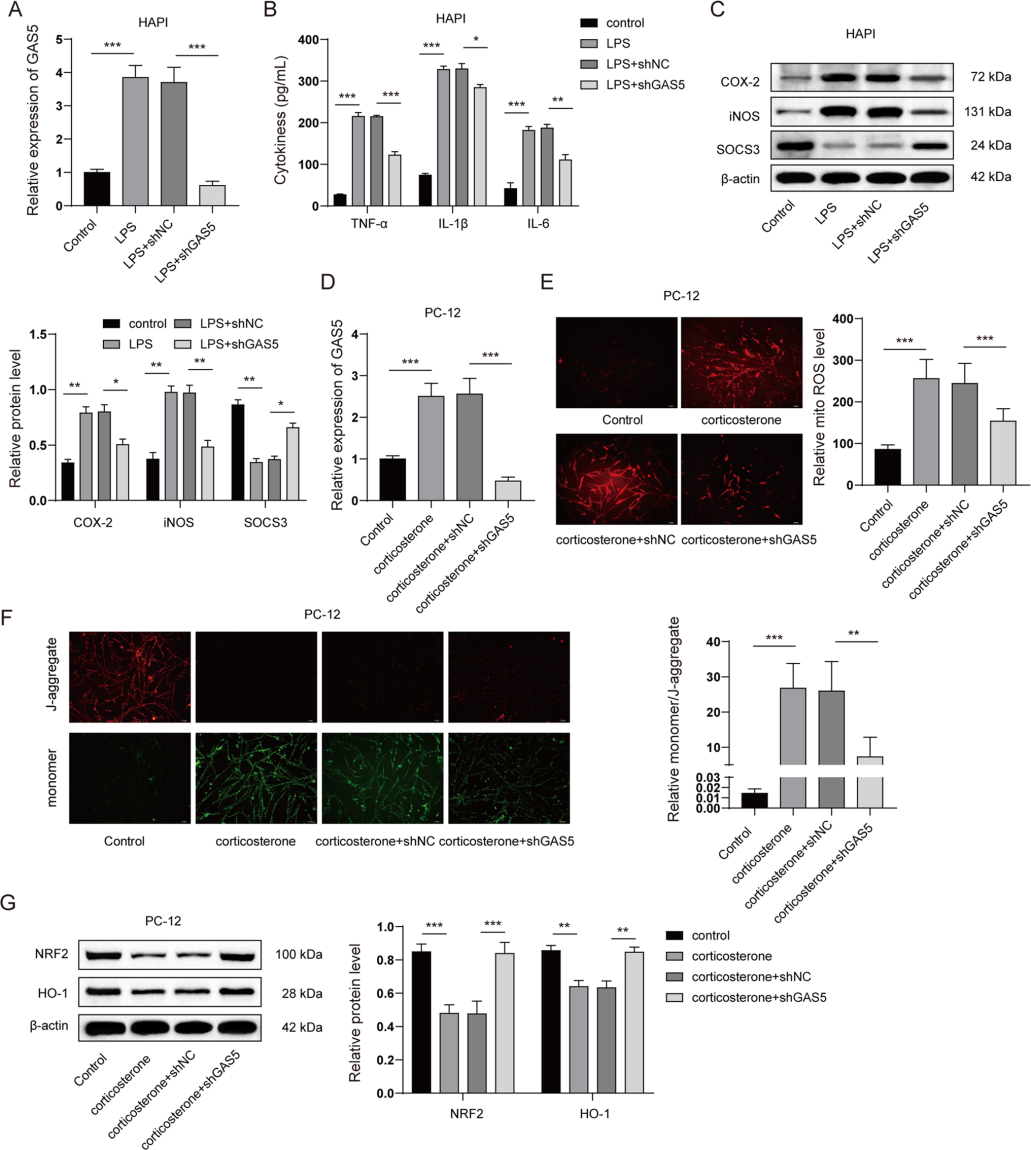
**GAS5 knockdown alleviated microglial activation and neuronal mitochondria dysfunction in vitro.** **A** and **B** Rat microglia cell line HAPI was transfected with GAS5 shRNA and 48 h after transfection, HAPI cells were stimulated with LPS (1 μM) for 24 h. **A **The expression of GAS5 was detected by qRT-PCR. **B** The release of pro-inflammatory cytokines was detected by ELISA. **C** The protein levels of COX-2, iNOS, and SOCS3 in HAPI cells were detected by western blots. **D** to **G** PC-12 cells were transfected with GAS5 shRNA and 48 h after transfection, PC-12 cells were subjected to corticosterone (400 μM) stimulation for 24 h. **D** The expression of GAS5 was detected by qRT-PCR. **E** Mitochondrial ROS in PC-12 cells was detected by the mitoSOX kit. **F** Mitochondrial membrane potential in PC-12 cells was detected by the JC-1 assay. **G** The expression of NRF2 and HO-1 in PC-12 cells was detected by western blots. *P < 0.05; **P < 0.01; ***P < 0.001

The cell treatment of the experiments is depicted in Figs. 6 and 7 and was as follows: for the LPS/corticosterone + Rg1 group, the cells were pre-treated with LPS (1 μg/mL, to establish an inflammatory stress model) or corticosterone (400 μM, to establish an oxidative stress model) for 2 h, followed by co-incubation with Rg1 (5 μM, 10 μM, 20 μM) for 24 h. For the LPS/corticosterone + shRNA group, the cells were transfected with lentivirus for 48 h and then washed with cold PBS buffer, followed by incubation with LPS or corticosterone for 24 h. Corticosterone (HY-B1618) was purchased from MedChemExpress and LPS (L5293) from Sigma-Aldrich.

### Sucrose Preference Test

The sucrose preference test (SPT) was performed for 24 h on the last day after CRS modeling. Two bottles of water were offered to the rats, one containing fresh water and the other containing 1% sucrose, and the positions of the two bottles were switched once to avoid the (left/right) position preference. The sucrose preference index was calculated as sucrose consumption/total liquid consumption × 100%.

### Open Field Test

The open field test (OFT) was performed after SPT. Briefly, the rats were placed at the center of an open field box (100 × 100 × 40 cm^3^) with 16 similar small grids drawn at the bottom. After 1 min of adaptation, the number of rearing and crossing that occurred within 5 min was recorded. The chamber was cleaned and wiped with 75% alcohol after each rat was tested.

### Forced Swim Test

The forced swim test (FST) was performed the day after performing OFT. For this purpose, a cylindrical vessel (measuring 50 cm in height and 20 cm in diameter) was used for FST. The vessel was filled with water (up to 30-cm high) at approximately 23 °C. Then, the rats were placed in water, and the time of immobility in 5 min (with nose above water) was recorded for each rat. Animal immobility was defined as staying still or making small movements in the water to keep the head above the water. The tested rats were then dried and returned to their cage at the end of each test.

### Western Blotting

Samples from the hippocampus tissues (15 mg) and 1 mL of RIPA lysis buffer (Beyotime, Shanghai) were added to a 2-mL EP tube. The solution of the EP tube was homogenized and centrifuged at 12,000 × *g* at 4 °C for 10 min, and the supernatant was collected. Samples from HAPI or PC-12 cells were given the following treatment: The treated cells (covering 80% area of the cell culture dish) were lysed in RIPA and centrifuged to obtain the supernatant. BCA method was used to determine the protein concentration. Proteins were then separated by SDS-PAGE (30 μg/lane) and then transferred onto a PVDF membrane. Skim milk (5%) dissolved in TBS was added to cover the membranes. After 2 h, the milk solution was discarded, and a primary antibody was incubated with the protein at 4 °C overnight. Next, the primary antibody was removed, and the membranes were washed with TBST and incubated with secondary antibody for another 1 h. Later, the protein bands were visualized by chemiluminescent immunoassay and quantified by Image J software. The following antibodies were used in the study: anti-COX-2 antibody (No.360120, Cayman), anti-iNOS antibody (ab178945, Abcam), anti-SOCS3 antibody (ab16030, Abcam), anti-NRF2 antibody (#33649, Cell Signalling Technology), anti-HO-1 antibody (#82206, Cell Signalling Technology), anti-EZH2 antibody (ab283270, Abcam), and anti-beta-actin (ab8226, Abcam).

### ELISA Assay for IL-1β, TNF-α, and IL-6

Whole blood samples of rats were obtained through eyeball extraction and stored at room temperature for 2 h, followed by centrifugation at 1500 × *g* for 10 min to collect the serum. The levels of IL-1β, TNF-α, or IL-6 were detected by using the enzyme-linked immunoassay (ELISA) assay kit (CUSABIO, Wuhan) with serum or hippocampus tissue lysate. Briefly, 50 μL of the samples or standards were added to each well, followed by the addition of a 50-μL of antibody cocktail that was incubated at 37 °C for 1 h. At the end of the incubation, the mixture was discarded, and the plate was washed with PBS. The TMB development solution was added for 10 min. Finally, 100 µL of the stop solution was added, and the optical density (OD) at 450 nm was recorded with a luminometer.

### ATP Production Assay

The ATP Assay Kit (Biotime, Shanghai) was used to test the production of ATP in the hippocampus tissues. For this purpose, the standard curve and the ATP working solution were prepared as suggested by the manufacturer’s instructions. A 50 μL of harvested hippocampus tissue lysate was added to each testing well, followed by the addition of 100 μL of ATP working solution to react for 3–5 min, followed by recording the OD value and calculation of the ATP concentration concerning the standard curve.

### Observation of Mitochondrial Morphology

The hippocampal tissues (< 1mm^3^) were fixed with 2.5% glutaraldehyde for 2 h, followed by washing with BPS and fixation with 1% osmic acid for 2 h and a final rinse with PBS. Gradually, the tissue was dehydrated with different concentrations of ethyl alcohol and acetone and then embedded with acetone solution overnight. After fixation, the tissue was cut into 50-nm slices and stained with 3% uranium acetate and lead citrate, and the mitochondrial morphology was then observed under a transmission electron microscope.

### Immunofluorescence Assay

The paraffin sections of the hippocampal tissues were subjected to an immunofluorescence (IF) assay. Briefly, the sections were washed with TBS containing 0.025% Triton X-100 and then incubated with 10% FBS for 2 h at room temperature, followed by incubation with anti-Iba1 antibody (1:100, Abcam, ab178847) at 4 °C overnight. The sections were then incubated with a fluorescent secondary antibody and DAPI in the dark and photographed under a fluorescence microscope.

### Mitochondrial DNA Isolation Procedure

Mitochondrial DNA (mtDNA) was integrity isolated using the mtDNA Isolation Kit (BioVision) according to the manufacturer’s instruction. Briefly, homogenized hippocampus tissue samples were centrifuged at 1200 × *g* for 10 min at 4 °C, and the supernatant was centrifuged at 10,000 × *g* for 15 min at 4 °C, after which the mitochondria were isolated in the precipitation. Enzyme B Mix was added to the mitochondrial extract and incubated at 50 °C in a water bath for 60 min until the solution became clear. The DNA pellet was finally washed with ethanol to obtain mtDNA for the subsequent qPCR test.

### Mitochondrial ROS Production Assay

The MitoSOX Red Mitochondrial Superoxide Indicator Kit (Invitrogen) was used to examine the mitochondrial ROS production. For this, a 5-μM of the working solution was prepared and added to treat the PC-12 cells for 10 min at 37 °C away from direct light. Then, the stained cells were mounted in a warm buffer for subsequent imaging.

### Mitochondrial Membrane Potential Assay

To determine the mitochondrial membrane potential, the JC-1 stain (JC-1 kit, Invitrogen) was diluted to 2 μg/mL concentration first. The treated PC-12 cells were harvested and washed with PBS and then incubated with the JC-1 stain working solution. The excess dye was washed off after 15 min, and the fluorescence intensity of the red and green color was observed under a fluorescence microscope.

### Quantitative Real-Time PCR

Total RNA was extracted from the tissue homogenate or cells using a TRIzol reagent (Invitrogen), chloroform, isopropanol, and 75% ethanol. After RNA purification, DEPC water was used to dissolve the extract to determine the concentration. For reserve transcription to synthesize cDNA, 1000 ng of the RNA was added to Takara RT reagent (Japan) and allowed to react at 37 °C for 15 min for 3 cycles and then at 85 °C for 5 s. Finally, 2 μL of the cDNA solution was applied for qRT-PCR using the SYBR Premix Kit (Takara) according to the manufacturer protocol. The reaction system was carried out at 95 °C for 30 s, 95 °C for 5 s, and 60 °C for 34 s for 40 repeats. The quantification of the target RNA level was calculated through the 2^–ΔΔCT^ method. The primer sequences were as follows (5′-3′): GAS5 forward GAGTGGGTGGGAAGTCTGAA, reverse GAGTGGGTGGGAAGTCTGAA; SOCS3 forward GCCTCAAGACCTTCAGCTCCAAG, reverse CGGTTACGGCACTCCAGTAGAATC; NRF2 forward GCCTTCCTCTGCTGCCATTAGTC, reverse TGCCTTCAGTGTGCTTCTGGTTG; GAPDH forward GTGGACCTCATGGCCTACAT, reverse TGTGAGGGAGATGCTCAGTG.

### RNA-Binding Protein Immunoprecipitation

The RIP Immunoprecipitation Kit (Millipore, USA) was used for the RNA-binding protein immunoprecipitation (RIP) experiment. HAPI or PC-12 cells were collected and resuspended in 200 μL of the RIP lysis with protease inhibitor and RNase inhibitor and then centrifuged at 14,000 × *g* at 4 °C for 10 min, and then, the supernatant was aspirated into another EP tube. In addition, protein A/G magnetic beads were washed and resuspended in 100 μL of RIP wash buffer, followed by incubation with 5 μL of EZH2 antibody (Abcam, ab283270) or IgG and rotated for 1 h at room temperature. Then, the EP tubes containing magnetic beads were placed on the magnet to retain the precipitation at the bottom. Next, 900 μL of RIP immunoprecipitation buffer and 100 μL of the cell lysate were added to the tube for incubation with the bead–antibody complex at 4 °C for rotation for 3 h. The next day, 150 μL of proteinase K buffer was added to each tube, and the resultant solution was mixed at 55 °C for 30 min. Furthermore, the tube was transferred to the magnet, and the supernatant was collected and combined with phenol, chloroform, and isoamylol to extract RNA from the immunoprecipitation. Finally, the RNA samples were prepared for qPCR to verify the potential target relationship between the GAS5 and EZH2 proteins.

### RNA Pull-Down

Sense with T7 promoter and antisense of GAS5 were synthesized, and in vitro transcription was subjected using respective primers by mMESSAGE mMACHINE kit (Ambion, USA). RNA size was detected by agarose gel. According to the manufacturer’s instruction, the biotin-labeled RNA (Bio-RNA) was prepared using an RNA 3′-end desthiobiotinylation kit (Thermo). The labeled RNA was purified immediately, followed by incubation with 50 μL magnetic beads at room temperature for 1 h rotation. The HAPI or PC-12 cells lysate mixed with 200 U/mL of RNase inhibitor and incubated with the magnetic beads overnight at 4 °C. Finally, the mixture was replaced with the magnet, and the beads were rinsed with the cell lysis buffer to collect the target protein, followed by western blotting.

### Chromatin Immunoprecipitation-qPCR (CHIP-qPCR)

The HAPI or PC-12 cells were washed with cold PBS and treated with 1% formaldehyde for 10 min to fix reversible protein-DNA cross-link and then mixed with glycine (125 mM) to quench the excess formaldehyde at room temperature. Subsequently, the cells were scraped and incubated with SDS lysis buffer containing proteinase inhibitor, then sonicated to cleave chromatin into a small fragment. Thus, after centrifugation, the supernatant was incubated with ChIP dilution buffer, 50 × PIC, and magnet beads with EZH2 or H3K27me3 antibody (ab6002, Abcam) or IgG prepared in advance. The next day, the immunoprecipitation complex was washed with the following solutions: low-salt wash buffer, high-salt wash buffer, LiCl, and wash buffer once and with TE buffer twice, after which the complex was eluted with a 10% SDS and 100 uL/M NaHCO_3_ dissolved in a ddH_2_O for overnight. The DNA was harvested using the Gene Elute kit (Merck) according to the protocol and analyzed by the qPCR assay.

### Statistical Analysis

GraphPad Prism 8.0 was used for data analysis. The data were recorded from no less than three independent experiments and expressed as mean ± SD. Student’s *t* test or one-way ANOVA was applied to compare the statistical difference, and *P* ≤ 0.05 was considered to indicate statistical significance.

## Results

### Rg1 Ameliorated Depression-Like Behaviours in CRS-Induced Rats

We first investigated the anti-depressive effect of Rg1 on CRS-induced rats. An open field test revealed that CRS treatment notably reduced the rearing and crossing counts. However, the injection of Rg1 or escitalopram (positive control) restored the rearing and crossing counts (Fig. 1A and Fig. 1B). Sucrose preference test revealed that Rg1 and escitalopram recovered CRS reduced sucrose consumption (Fig. 1C). Additionally, the forced swim test illustrated that CRS increased the immobility time of rats, whereas the injection of Rg1 or escitalopram reduced it (Fig. 1D). These results confirmed that Rg1 treatment alleviated CRS-induced depression-like behaviours in CRS rats.

### Rg1 Abrogated Activation of Microglial and Attenuated Mitochondrial Dysfunction in CRS-Induced Rats

Overactivation of microglia is involved in the pathogenesis of depression. We next investigated whether Rg1 affected microglial activation in CRS-induced rats. As shown in Fig. 2A, CRS increased IBA-1 positive cells (a microglia marker) in the hippocampus, whereas Rg1 or escitalopram treatment substantially decreased the IBA-1 positive cells. Moreover, we also discovered that the levels of pro-inflammatory cytokines (TNF-α, IL-1β, and IL-6) in the serum and hippocampus were dramatically elevated by CRS treatment. However, Rg1 or escitalopram reduced the levels of these pro-inflammatory cytokines (Fig. 2B and Fig. 2C). Mitochondrial dysfunction was also commonly observed in depression. In the present study, we found that the ATP level notably decreased in the CRS-induced group. Rg1 or escitalopram partly restored the ATP level (Fig. 2D).

Additionally, the transmission electron microscopy (TEM) analysis of mitochondrial morphology revealed mitochondrial injury and a decrease in the length–width ratio of mitochondria in the CRS group. However, Rg1 or escitalopram alleviated mitochondrial injury and restored the length–width ratio of mitochondria (Fig. 2E). Moreover, CRS decreased the copy number of mitochondrial DNA (mtDNA), whereas Rg1 or escitalopram recovered the copy number of mtDNA (Fig. 2F). To investigate Rg1 regulation of microglial activation and mitochondrial function in depression, we analyzed the expression of several proteins that might be involved in the progress of inflammatory response and oxidative stress. As shown in Fig. 2G, the results illustrated that Rg1 and escitalopram restored the expressions of SOCS3 and NRF2, which were reduced in CRS-treated mice. SOCS3 and NRF2 were negatively regulated by EZH2-mediated epigenetic suppression [28, 29]. We also tested whether Rg1 influences the expression of EZH2. The results indicated that EZH2 was elevated in the CRS rat model. However, Rg1 treatment failed to exhibit a significant change in the expression of EZH2. GAS5 was reported as a lncRNA that enhanced the suppressive function of EZH2 on gene transcription [30]. Hence, we also detected the expression of GAS5. Interestingly, we observed that Rg1 treatment notably decreased GAS5 expression induced by CRS (Fig. 2H). Collectively, these results revealed that Rg1 abrogated microglial activation and attenuated mitochondrial dysfunction in CRS-induced rats.

### GAS Knockdown Ameliorated Depression-Like Behaviours in CRS-Induced Rats

We next investigated the anti-depressive effect of GAS5 knockdown on CRS rats. In vivo GAS5 knockdown was achieved by injecting lentivirus-packaged GAS5 short hairpin RNA (shRNA) (Lenti-shGAS5) into the rat brain. qPCR revealed that CRS notably elevated the expression of GAS5, whereas the injection of Ad-shGAS5 significantly repressed the expression of GAS5 in the hippocampus (Fig. 3A). The open field test illustrated that CRS reduced the rearing and crossing counts, whereas the injection of Ad-shGAS5 restored the number of rearing and crossing counts (Fig. 3B and Fig. 3C). Sucrose preference test revealed that GAS5 knockdown recovered the sucrose consumption reduced by CRS (Fig. 3D). Moreover, the forced swim test showed that CRS increased the immobility time of rats, whereas GAS5 knockdown reduced the immobility time (Fig. 3E). These results confirmed that GAS5 knockdown alleviated CRS-induced depression-like behaviours in rats.

### GAS5 Knockdown Suppressed Microglial Activation and Attenuated Mitochondrial Dysfunction in CRS-Induced Rats

We next investigated that whether GAS5 knockdown affected microglial activation and mitochondria dysfunction in CRS-induced rats. As shown in Fig. 4A, CRS increased IBA-1 positive cells in the hippocampus, whereas GAS5 knockdown decreased IBA-1 positive cells. Moreover, the levels of pro-inflammatory cytokines, including TNF-α, IL-1β, and IL-6, in the serum and hippocampus were dramatically elevated by CRS treatment, whereas GAS5 knockdown decreased the level of the pro-inflammatory cytokines (Fig. 4B and Fig. 4C). In terms of mitochondrial dysfunction, the ATP level notably reduced in the CRS-induced group, which was restored by GAS5 knockdown (Fig. 4D). Additionally, TEM analysis revealed the mitochondrial injury and a decrease in the mitochondrial length–width ratio in the CRS group. However, GAS5 knockdown ameliorated mitochondrial injury and recovered the length–width ratio of mitochondria (Fig. 4E). Similarly, our results also suggested that GAS5 knockdown restored the copy number of mtDNA decreased by CRS treatment (Fig. 4F). We also tested whether GAS5 regulated the expression of NRF2, SOCS3, and EZH2 in the CRS model. As shown in Fig. 4G, the results illustrated that GAS5 restored the expressions of SOCS3 and NRF2, which were suppressed in CRS-treated mice. However, the GAS5 knockdown failed to reveal a significant influence on the expression of EZH2. Collectively, these results demonstrated that GAS5 knockdown suppressed microglial activation and attenuated mitochondrial dysfunction in CRS-induced rats.

### Rg1 Exerted a Protective Effect on the Rat CRS-Induced Depression Model Downregulating GAS5

To investigate whether GAS5 mediated the protective effect of Rg1 in depression, we injected lentivirus-packaged GAS5 overexpressing plasmid (Lenti-GAS5) into the rat brain before Rg1 treatment and modeling. As shown in Fig. 5A, the overexpression of GAS5 substantially elevated the Rg1-reduced expression of GAS5 in the hippocampus. Behavioural experiments suggested that Rg1 alleviated depression-like behaviours, as indicated by increased rearing counts, crossing counts, sucrose consumption, and decreased immobility time. However, the overexpression of GAS5 partly compromised the protective effect of Rg1 on depression-like behaviours (Fig. 5B to Fig. 5E). Moreover, GAS5 reversed the effect of Rg1 on microglial activation and mitochondrial dysfunction. Immunofluorescence revealed that Rg1 reduced IBA-1 positive cells, whereas tGAS5 overexpression reversed this result (Fig. 5F). Additionally, the overexpression of GAS5 compromised the Rg1-induced increase in the ATP level, length–width ratio, and mtDNA copy number (Fig. 5G to I). Rg1 consistently increased the expression of SOCS3 and NRF2 in the depression model, whereas the GAS5 overexpression compromised this effect, as indicated by a notable decrease in the expression of SOCS3 and NRF2 (Fig. 5J). Overall, our results indicated that GAS5 mediated the protective effect of Rg1 in depression.

### Rg1 Alleviated Microglial Activation and Neuronal Mitochondria Dysfunction In Vitro

To validate the findings of our in vivo study, we next investigated the effect of Rg1 (5 μM, 10 μM, 20 μM) on microglial activation and neuronal mitochondrial dysfunction in vitro. Rat microglia cell line HAPI was stimulated with LPS to mimic microglial activation in vitro. Enzyme-linked immunoassay revealed that LPS dramatically promoted the release of pro-inflammatory cytokines, whereas Rg1 treatment suppressed the pro-inflammatory cytokine levels in the cell culture supernatant (Fig. 6A). Interestingly, Rg1 treatment alone also decreased the level of TNF-α and IL-1β while showing no significant effect on IL6. Moreover, LPS stimulation significantly elevated the expression of the pro-inflammatory proteins COX-2 and iNOS and decreased the expression of SOCS3 in HAPI cells. Consistent with our expectations, Rg1 treatment reduced COX-2 and iNOS expressions and restored the expression of SOCS3 in LPS-stimulated HAPI cells. Rg1 treatment alone also led to a decrease of the basal expression of COX-2 and iNOS and upregulated SOCS3 (Fig. 6B). PC-12 cells were subjected to corticosterone stimulation to mimic in vitro mitochondrial dysfunction in depression. As shown in Fig. 6C, corticosterone stimulation notably increased the level of mitochondrial ROS in PC-12 cells, whereas Rg1 treatment sharply reduced the level of mitochondrial ROS. Mitochondrial membrane potential was detected using the JC-1 assay. The results suggested that corticosterone stimulation lowered the mitochondrial membrane potential (MtMP) (Δψm), as indicated by the JC-1 aggregate-monomer ratio. Interestingly, the Rg1 treatment substantially restored the MtMP (Fig. 6D). Rg1 treatment alone did not show a notable influence on the basal ROS and MMP (Fig. 6C and 6D). Moreover, the expression of NRF2 and its downstream protein HO-1 were suppressed in the presence of corticosterone, which was recovered by Rg1 treatment. Rg1 treatment alone also led to an increase of the basal expression of NRF2 and HO-1 (Fig. 6E). Additionally, we validated that LPS and corticosterone significantly upregulated the level of GAS5 while Rg1 treatment led to a notable decrease of GAS5. Rg1 treatment alone also attenuated the basal expression of GAS5 (Fig. 6F and 6G). Collectively, we demonstrated that Rg1 alleviated microglial activation and neuronal mitochondrial dysfunction in vitro.

### GAS5 Knockdown Alleviated Microglial Activation and Neuronal Mitochondrial Dysfunction In Vitro

We next investigated the effect of GAS5 knockdown on microglial activation and neuronal mitochondrial dysfunction in vitro. qPCR revealed that GAS5 shRNA sharply diminished the expression of GAS5 upon LSP stimulation (Fig. 7A). Moreover, the results revealed that LPS notably enhanced the release of pro-inflammatory cytokines in HAPI cells, whereas GAS5 knockdown suppressed the level of pro-inflammatory cytokines in the cell culture supernatant (Fig. 7B). LPS stimulation also elevated the expression of COX-2 and iNOS and reduced SOCS3 expression. Interestingly, GAS5 knockdown reduced COX-2 and iNOS expressions but restored the expression of SOCS3 (Fig. 7C). In terms of mitochondrial dysfunction, as shown in Fig. 7D and E, corticosterone stimulation substantially increased the level of GAS5 and mitochondrial ROS in PC-12 cells, whereas GAS5 knockdown significantly attenuated the level of GAS5 and mitochondrial ROS. The JC-1 assay findings indicated that corticosterone stimulation suppressed the MtMP, as noted in the JC-1 aggregate-monomer ratio. However, the GAS5 knockdown substantially elevated the MtMP (Fig. 7F). Our results also revealed that the expression of NRF2 and its downstream protein HO-1 were decreased by corticosterone stimulation, which was recovered by GAS5 knockdown (Fig. 7G). Collectively, these results suggested that GAS5 knockdown alleviated microglial activation and neuronal mitochondrial dysfunction in vitro.

### GAS5 Suppressed the Expression of NRF2 and SOCS3 Via EZH2-Mediated Epigenetic Repression

**Fig. 8:**
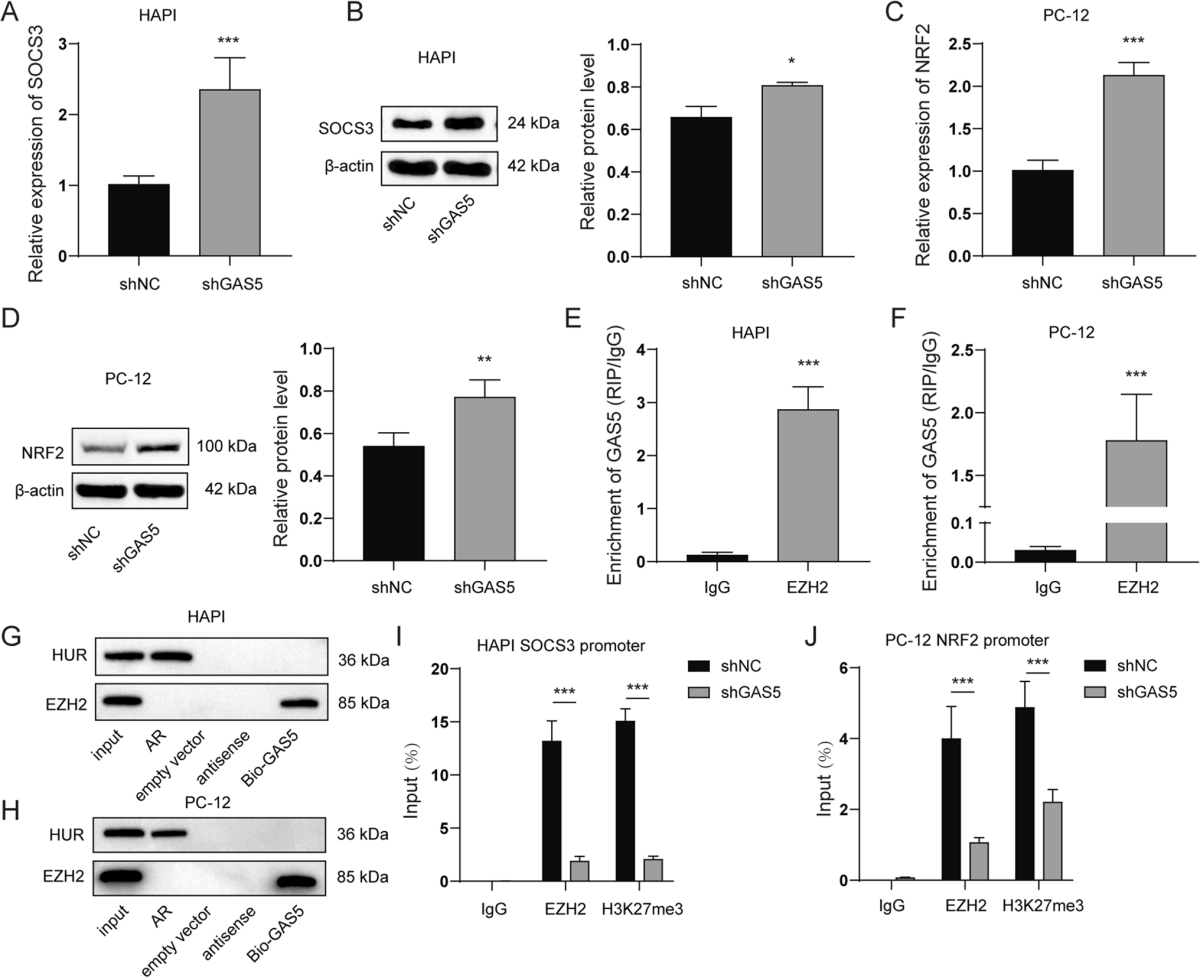
GAS5 suppressed the expression of NRF2 and SOCS3 via EZH2-mediated epigenetic repression. HAPI cells and PC-12 cells was transfected with GAS5 shRNA. 48 after transfection, followed experiments were performed. **A** and **B** The level of SOCS3 in HAPI cells was detected by qPCR (**A**) and western blots (**B**). **C** and **D** The level of NRF2 in PC-12 cells was detected by qPCR (C) and western blots (**D**). **E** and **F** RIP was performed to validate binding between GAS5 and EZH2. **G** and **H** RNA pull-down assay was performed to validate binding between GAS5 and EZH2. **I** CHIP-qPCR was performed to validate the enrichment of EZH2 and H3K27me3 in the promoter region of SOCS3 in HAPI cells. **J** CHIP-qPCR was performed to validate the enrichment of EZH2 and H3K27me3 in the promoter region of NRF2 in PC-12 cells. *P < 0.05; **P < 0.01; ***P < 0.001

We next investigated the regulation of the expression of SOCS3 and NRF2 by GAS5. GAS5 was silenced by shRNA in HAPI and PC-12 cells. Consistent with our expectations, the expression of SOCS3 in HAPI cells (Fig. 8A and B) and NRF2 in PC-12 cells (Fig. 8C and D) was upregulated upon GAS knockdown. GAS5 mediated gene suppression by binding with EZH2 and facilitating EZH2-mediated epigenetic repression [30]. In the present study, RNA immunoprecipitation (RIP) and RNA pull-down assays revealed GAS5 directly bound with EZH2 in HAPI and PC-12 cells (Fig. 8E to 8H). CHIP-qPCR was performed to validate the enrichment of EZH2 and H3K27me3 in the promoter region of SOCS3 or NRF2, which revealed that GAS5 knockdown significantly attenuated the enrichment of EZH2 and H3K27me3 in the promoter region of SOCS3 in HAPI cells (Fig. 8I). Similarly, GAS5 knockdown reduced the enrichment of EZH2 and H3K27me3 in the promoter region of NRF2 in PC-12 cells (Fig. 8J). Collectively, these results indicated that GAS5 might suppress the expression of NRF2 and SOCS3 via EZH2-mediated transcriptional repression.

### SOCS3 or NRF2 Knockdown Compromised the Protective Effects of Rg1 in HAPI and PC-12 In Vitro Model

**Fig. 9:**
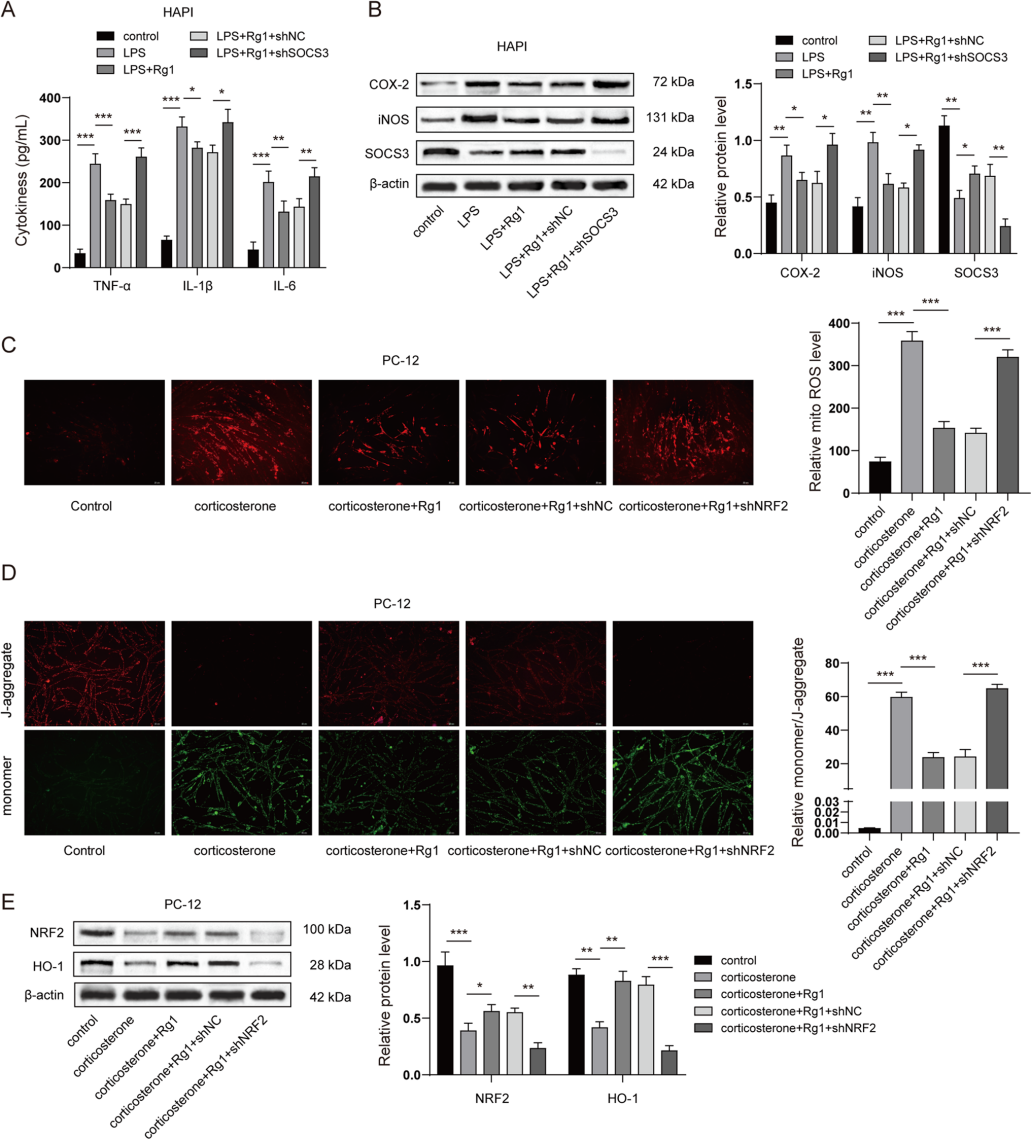
SOCS3 or NRF2 knockdown compromised the protective effects of Rg1 in HAPI and PC-12 in vitro model. **A** and **B** HAPI cells were transfected with SOCS3 shRNA. Forty-eight hours after transfection, the cells were stimulated with LPS (1 μM) for 2 h, followed by co-incubation with Rg1 (5 μM, 10 μM, 20 μM) for a total 24 h LPS and Rg1 (**A**). The release of pro-inflammatory cytokines in supernatant of HAPI cells was detected by ELISA. **B** The protein level of COX-2, iNOS, and SOCS3 in HAPI cells was detected by western blots. **C** to **E** PC-12 cells were transfected with NRF2 shRNA. Forty-eight hours after transfection, the cells were stimulated with corticosterone (400 μM) for 2 h, followed by co-incubation with Rg1 (5 μM, 10 μM, 20 μM) for a total 24 h subjected to corticosterone stimulation and Rg1 treatment. **C** Mitochondrial ROS in PC-12 cells was detected by the mitoSOX kit. **D** Mitochondrial membrane potential in PC-12 cells was detected by the JC-1 assay. **E** The expression of NRF2 and HO-1 in PC-12 cells was detected by western blots. *P < 0.05; **P < 0.01; ***P < 0.001

To validate NRF2 or SOCS3 directly mediated the protective effect of Rg1 on mitochondrial dysfunction and repress microglial activation in vitro model, we applied shRNA to knockdown SOCS3 in HAPI cells and NRF2 in PC-12 cell before stimulation and Rg1 treatment. As shown in Fig. 9A, Rg1 potentially reduced LPS induced releasing of cytokines while SOCS3 knockdown reversed the effect of Rg1 on cytokine release. Consistently, we observed that SOCS3 compromised the reduction of COX-2 and iNOS by Rg1 (Fig. 9B). In PC-12 cells, NRF2 knockdown was verified to reverse the protective effect of Rg1 on intracellular ROS and mitochondrial potential (Fig. 9C and 9D). Western blots in Fig. 9E also indicated that NRF2 knockdown sharply dropped the expression of HO-1 and NRF2, which was restored by Rg1 in corticosterone stimulated PC-12 cells. Collectively, these results suggested that Rg1 alleviated microglial activation and neuronal mitochondrial dysfunction at least partly dependent on upregulation of SOCS3 and NRF2.

## Discussion

Depression is a severe mental disorder characterized by depressed mood, retardation of thoughts, and loss of voluntary activity. For a long time, the pathogenesis of depression has remained unclear; various factors, including neuroendocrine, neuroimmunology, genetics, and social influences, may be involved in its pathogenesis [2]. In the present study, our results revealed the anti-depressive effect of Rg1 in the depression model. Rg1 treatment attenuated microglial activation and improved mitochondrial dysfunction, thereby alleviating depression-like behaviours by downregulating lncRNA GAS5. GAS5 inhibition exhibited a similar protective effect of Rg1 treatment. Mechanically, GAS5 inhibition upregulated the expressions of NRF2 and SOCS3, which EZH2 and GAS5 suppressed. In the present study, the mechanism of the anti-depressive effect of RG1 was deeply explored, providing a theoretical basis for the possible application of RG1 in the treatment of depression.

The anti-inflammatory effects of Rg1 have been validated in various neurological diseases. Rg1-ameliorated chemotherapy-induced cognitive dysfunction by suppressing microglia-mediated neuroinflammation [31]. In Parkinson’s disease, Rg1 reduces LPS-induced neuroinflammation via G-protein coupled oestrogen receptor [32]. Moreover, Zhang et al. demonstrated that Rg1 abrogated microglial activation in the post-traumatic stress disorder model [33]. In the present study, we verified the anti-inflammatory role of Rg1 in the depression model. The treatment of Rg1 reduced IBA-1 positive cell numbers in the hippocampus and abrogated the release of pro-inflammatory cytokines. This finding was consistent with previous studies, which confirmed the anti-inflammatory effect of Rg1 in the depression model [20, 34]. Additionally, Rg1 is involved in the regulation of mitochondria function. Xu et al. suggested that Rg1 improved mitochondrial function by suppressing oxygen–glucose deprivation-induced injury in astrocytes [35]. Another report revealed that Rg1 repressed isoflurane-induced caspase-3 activation by preventing mitochondrial dysfunction [36]. As mentioned previously, mitochondrial dysfunction was generally found in the depression model. In the present study, we first validated the beneficial effect of Rg1 on mitochondria in the depression model.

Some studies have revealed that Rg1 exerts its function via the regulation of lncRNA. A previous report has suggested that Rg1 inhibits high glucose-induced fibrosis by regulating lncRNA RP11-982M15.8 [37]. To the best of our knowledge, we are the first to validate that Rg1 regulated lncRNA GAS5. A functional study revealed that GAS5 exhibits a protective effect in the depression model by downregulating GAS5 expression. The inhibition of GAS5 revealed an anti-inflammatory impact on spinal cord ischemia reperfusion [38] and in oxidative stress induced by oxidized low-density lipoprotein [39]. Moreover, GAS5 is involved in mitochondria-mediated apoptosis, indicating that GAS5 might regulate mitochondrial function [40]. In the present study, we confirmed that the inhibition of GAS5 alleviated microglial activation and mitochondrial dysfunction, thereby ameliorating depression-like behaviours in the depression model. This finding was consistent with the previous study [27].

Moreover, the present study revealed the detailed mechanism of GAS5 in depression. We found that GAS5 was directly bound with EZH2 and mediated the suppression of NRF2 and SOCS3 by EZH2. This finding deepened the understanding of the mechanism by which GAS5 exhibits its detrimental role in depression. However, we failed to validate how Rg1 regulated GAS5 and whether Rg1 could regulate other lncRNA involved in the pathology of depression. We must focus on this obstacle in our further investigation.

The zeste homolog 2 enhancer (EZH2) is the major subunit of polycomb repressive complex 2 (PRC2). EZH2 suppresses the downstream gene expression via the methylation of histone 3 on lysine 27 (H3K27me3) in the promoter of the target gene [41]. The function of EZH2 is generally regulated by lncRNA and circular RNA. GAS5 inhibits the level of ATP-binding cassette transporter A1 (ABCA1) by directly binding with EZH2 and facilitating the methylation of H3K27me3 modification in the promoter region of ABCA1 [42]. This finding was supported by other studies, which indicated that GAS5 suppresses the expression of MMP9 by recruiting EZH2 to the promoter region of MMP9 [30]. Consistently, in the present study, we also validated that GAS5 facilitated the function of EZH2 in the suppression of NRF2 and SOCS3. GAS5 knockdown decreased EZH2 and H3K27me3 expressions in the promoter region of NRF2 and SOCS3 and thereby rescued the expression of NRF2 and SOCS3. However, other studies have revealed a contentious relationship between GAS5 and EZH2. A study by Zhu et al. suggested that GAS5 suppresses Th1 differentiation by inhibiting the expression of EZH2 [43]. Another study further validated that GAS5 decreased the expression of EZH2 via recruiting E2F4 to the promoter region of EZH2 [44]. This contrary conclusion might be due to the different regulations of GAS5 on EZH2 in various diseases. EZH2 mediates neuroinflammation and depression-like behaviours [45], which is in line with the findings of the present study. However, the functional role of EZH2 in depression should be further investigated in future work.

In the present study, we revealed the expression of two downstream genes, NRF2 and SOCS3, was inhibited by the GAS5/EZH2 axis in the depression model. The epigenetic suppression of SOCS3 by EZH2 was validated in a previous study [28], which was also supported by the findings of our present study. SOCS3 negatively regulates microglial activation in neuropathic pain [46]. Interestingly, the inhibition of EZH2 in a depression model elevated the expression of SOCS3 [45], which suggested that SOCS3 might regulate microglial activation in depression. However, more studies are warranted to confirm this hypothesis. NRF2 is a major transcription factor responsible for the antioxidant effect in cells [47]. NRF2 mediated the protective effect of adipose-derived mesenchymal stem cell (ADSC) [48] and melatonin [12] on depression-like behaviours. Previous work has also indicated that NRF2 has a protective effect on mitochondrial dysfunction [49]. Consistent with our findings, another research also validated that NRF2 was downregulated in a depression model and restored by Rg1 treatment [34]. Therefore, we speculated that Rg1/GAS5 regulates mitochondrial dysfunction in depression by regulating the expression of NRF2. More studies are warranted to validate this hypothesis.

In conclusion, we verified the protective role of Rg1 in a depression model. Rg1 attenuated microglial activation and improved mitochondrial dysfunction by downregulating the expression of GAS5. We also validated the anti-depressive function of GAS5 knockdown in our present study. Mechanically, GAS5 regulated microglial activation and mitochondrial dysfunction via the EZH2-mediated epigenetic suppression of NRF2 and SOCS3. Our result might provide novel insights into understanding the role of Rg1 in the treatment of depression.

## Data Availability

The datasets used or analyzed during the current study are available from the corresponding author on reasonable request.

## Abbreviations

CRS: Chronic restraint stress
SSRIs: Selective 5-HT reuptake inhibitors
CNS: Central nervous system
CMS: Chronic mild stress
ROS: Reactive oxygen species
GAS5: Growth arresting-specific 5
EZH2: Enhancer of zeste homolog 2
PRC2: Polycomb repressive complex 2
H3K27me3: Methylation of histone 3 on lysine 27
SOCS3: Suppressor of cytokine signaling 3
Nrf2: Nuclear factor E2-related factor 2
